# Clean, Pleasant, Governed: A Critical Discourse Analysis of *Bathing Without a Battle*

**DOI:** 10.1111/nin.70155

**Published:** 2026-08-02

**Authors:** Patricia Morris, Rose McCloskey, Janet Durkee‐Lloyd, Karla O'Regan

**Affiliations:** ^1^ Faculty of Nursing and Health Sciences University of New Brunswick Fredericton New Brunswick Canada; ^2^ Department of Gerontology St. Thomas University Fredericton New Brunswick Canada; ^3^ Department of Criminology St. Thomas University Fredericton New Brunswick Canada

**Keywords:** assisted bathing, autonomy, capacity, dementia care, governance, nursing education, resistance to care

## Abstract

*Bathing Without a Battle* is a widely circulated video training resource in residential dementia care, but the assumptions it advances about bathing, refusal, and appropriate nursing practice have received limited critical attention. This paper analyzes how the text constructs resistance to assisted bathing and how it frames legitimate nursing responses to that resistance. A three‐part Faircloughian critical discourse analysis is used, attending to social structures, processes of production and dissemination, and the textual features through which meaning is enacted. The bulk of the analysis focuses on genre, discourse, and style to examine how the video governs practice and moral reasoning. Findings indicate that the program positioned itself as an early form of clinical practice guideline by linking instructional conventions with the authority of academic research. It also illuminates the lexical choices used to portray cleanliness as a non‐negotiable objective, while clients' refusals are positioned as problems (of technique, discomfort, or pathology) to overcome rather than expressions of agency. Finally, the paper demonstrates how the video's emphasis on pleasure operates as a stylistic and moral device that shapes nurses as responsible for producing care that is both hygienic and affectively acceptable. The paper concludes by considering the ethical implications of this framing for autonomy in dementia care and the normalization of persuasion when care outcomes are predetermined.

**Trial Registration:** Clinical trial registration is not applicable, as this study did not involve a clinical trial

1

For many people with class, neurotypical, and able‐bodied privilege, bathing is a site of relaxation and body modification. It is an experience marked by low lighting or vigorous exfoliation (or both) that blurs the line between hygiene and pleasure. But this is not the experience of everyone who bathes. Bathing is among the most challenging activities of daily living (ADLs) for people living with dementia (Alzheimer's Association 2026; González‐Mella and Unzueta‐Arce 2025). It is a complex ADL, requiring the coordination of advanced physical and cognitive abilities (Shimokihara et al. 2022). People living with dementia may struggle with agnosia, apraxia, or balance difficulties, making it hard to distinguish familiar objects, perform motor sequences, or safely navigate the environment (Moinuddin et al. 2022). Bathing may trigger fear or defensive aggression, rooted in the body's fight‐flight‐freeze response to perceived threat (Lewington et al. 2024). What was once an invigorating ritual can become an exhausting and frightening experience for people living with dementia, and a challenging one for caregivers (Amato et al. 2021).

The literature on assisted bathing in dementia tends to focus on interventions that improve a client's cooperation with bathing. Music, pleasing photos, video‐simulated presence of a loved one, essential oils, and a host of other interventions have been trialed for their utility in decreasing responsive behaviors during bathing (see, e.g., Backhouse et al. 2020; Hammar et al. 2011; Kong et al. 2009; O'Connor et al. 2011). Like the researchers and clinicians who design and implement these strategies, we are deeply concerned about the experiences of people with dementia who decline or resist bathing. In particular, we are concerned by the persistent focus in research and practice on fixing the “problem” of clients' refusals that do not delve into the conditions that produce those refusals as a problem in the first place. Why are refusals to bathe constructed as a clinical or behavioral problem requiring intervention rather than as a meaningful expression of agency and distress?

In this paper, we concern ourselves with the ways that refusals of assisted bathing are discursively constructed as a problem in need of solving, and with the solutions that are proposed to address this problem. We undertake a critical discourse analysis (CDA) of a key “text” designed for educating caregivers on bathing innovations for people living with dementia: *Bathing Without a Battle: Creating a Better Bathing Experience for Persons With Alzheimer's Disease and Related Dementias* (Barrick et al. 2003). The video was released in 2003, and yet it is still widely cited as a gold standard in bathing education for frontline care providers (Hanson et al. 2023; Lorusso et al. 2022; Nguyen et al. 2023). The video is intended to persuade staff to change their approach to assisted bathing by promoting person‐centered bathing plans and techniques. It includes videos of actual encounters between clients and long‐term care (LTC) staff, with examples of techniques that prompted real resistance from clients and examples of techniques that were easily accepted by the same clients. The techniques demonstrated in the videos have been empirically tested (Gozalo et al. 2014; Hoeffer et al. 2006; Sloane et al. 2004). In recent years, the trials have been subject to scrutiny for their failure to protect the privacy of people living with dementia, and for failing to obtain their informed consent (Nix et al. 2023), and yet the strategies and the problem they purport to solve remain largely without critique.

The guiding research question for this analysis was: how does *Bathing Without a Battle* (BWAB) construct clients' resistance to care, and what appropriate nursing responses are intelligible in the context of residential dementia care?

## 2 Methodology

CDA provided the methodological framework for this. Broadly, discourse analytic methods examine patterns of “language‐in‐use” (Taylor 2001, 6), focusing on how language operates within social contexts to produce meaning. While approaches to discourse analysis vary across disciplines, they share a methodological interest in understanding language as social action rather than as an abstract system of rules. What distinguishes CDA from other discourse analytic traditions is its explicitly critical orientation. It seeks to uncover the ways that language maintains or challenges relations of power and inequity (Strauss and Feiz 2013). Rather than merely describing linguistic patterns, CDA asks how discursive practices shape social realities and sustain inequitable distributions of power (Fairclough 2003, 2010).

This analysis draws specifically on Norman Fairclough's approach to CDA, which conceptualizes discourse as both socially shaped and socially shaping. Fairclough's framework links textual analysis to broader social and institutional practices, emphasizing that language simultaneously represents, constructs, and legitimizes the social world. His approach is grounded in critical realism (Bhaskar 2008), which is a philosophical position that assumes a real world exists independently of our descriptions of it and acknowledges that our access to that reality is always mediated by discourse. From this perspective, discourse does not simply mirror reality but actively shapes how people talk, behave, and interact. Critical realism provides CDA with an ontological depth which pushes researchers to move beyond surface description of linguistics to examine how language reflects and reproduces relations of power and inequity and the institutions in which they take place. In this study, Fairclough's (2003) critical realist orientation supports an analysis of BWAB as a site where language participates in constructing the material realities of dementia care.

## 3 Methods

The data for this study consisted of the BWAB training DVD, which was obtained through the first author's professional practice as a Clinical Nurse Specialist in geriatrics at a small hospital specializing in geriatric medicine. The DVD had been used in staff orientation for several years and was identified at orientation to the role as a resource that is always included in training for new frontline staff. The first author's clinical and research background in gerontological nursing informed the selection of BWAB as a text of interest and shaped initial critical questions about how educational materials represent people living with dementia and the nurses who care for them. Beyond this local clinical context, BWAB has been and continues to be widely circulated in LTC, with the Pioneer Network identifying it as an “artifact of culture change” and an exemplar of client‐directed (Pioneer Network 2021a, 2021b). It continues to be cited in contemporary research literature as well (Hanson et al. 2023; Lorusso et al. 2022; Nguyen et al. 2023). This sustained presence across clinical, organizational, and academic contexts informed its selection as a rich text for examining the discursive construction of resistance to hygiene care in dementia practice.

The video was transcribed verbatim using automated transcription software (Otter.ai). The transcript was reviewed and corrected by the first author and independently verified against the original recording by the first and second authors to ensure accuracy and completeness. The finalized transcript was imported into MAXQDA (VERBI Software) for coding and data management. An audit trail was maintained throughout the analytic process, including memos documenting coding decisions, analytic reflections, and iterative category development.

Analysis was guided by Norman Fairclough's (2003) critical discourse analytic framework. The first and second authors (both nursing scholars) participated in the analytic process. The first author conducted initial coding and developed preliminary analytic categories. The second author reviewed coded segments, analytic memos, and emerging interpretations, and provided critical feedback. Discrepancies and analytic decisions were discussed and refined through dialogue with the entire research team, which consisted of a gerontologist and law professor with a speciality in CDA. The interdisciplinarity of the team contributed to analytic rigor and reflexivity.

Following Fairclough's framework, the team examined BWAB across three interrelated levels. The first was the level of social structures, which are the wider sociocultural contexts that give rise to texts and set out (at least partially) what it is possible to say at a particular point in history. The second was at the level of the individual text's production, distribution, and consumption. The third, and most robust for this analysis, was at the level of the text where genre, discourses, and styles are enacted. This multi‐level analysis enabled a critical examination of how the BWAB program participates in shaping professional understandings of resistance to care and delineates the moral and clinical boundaries of “good” nursing within dementia care.

## 4 Findings

### 4.1 Level 1: Social Structures

The BWAB training program was conceived by a group of nurse‐researchers at the University of North Carolina. Dr. Joanne Rader and Dr. Anne‐Louis Barrick envisioned a better world where people with dementia might receive more comfortable baths in residential care facilities and where frontline care providers might experience less verbal and physical resistance to their attempts to do their jobs. The problem this training program seeks to tackle is described like this on the program's website:

Each day hundreds of thousands of people with dementia are bathed against their will. Their overt or nonverbal refusals are often ignored, and they are removed without permission from their beds and wheelchairs and taken to an often cold, impersonal, frightening shower or tub room to be scrubbed down. As a result, the refusals escalate to verbal and physical resistance, and finally to combativeness. The experience is frustrating and dangerous to caregivers, who become the targets of hitting, spitting, biting, and verbal attacks by the person who they are only trying to help.

(“About Bathing” n.d., para. 1)

The problem, as the researchers set it out, is that unwanted bathing for people with dementia was an epidemic. Residents' refusals were routinely “ignored”; their consent was violated when they were “removed from their beds” and taken to “cold, impersonal, frightening” shower and tub rooms; they were “scrubbed down” like the pots in my kitchen, by frustrated caregivers who are “only trying to help” but are routinely subject to “attacks.” The lexical choices in this description of typical bathing practices in LTC homes are powerful, and they certainly offer support for the researchers' agenda of making bathing a “more humane, gentle experience” for clients (“About Bathing” n.d., para. 2).

Five interrelated social structures shaped the discursive conditions under which BWAB could be conceived, circulated, and received with high regard. The first was biomedical discourse. During the 1980s and 1990s, diverse cognitive, behavioral, and functional changes associated with multiple underlying diseases (e.g., Alzheimer's disease, vascular disease, Lewy body disease, and frontotemporal degeneration) were increasingly organized under the category of *dementia* and distinguished in popular discourse from so‐called normal ageing (Fletcher 2021; Fletcher and Maddock 2021). While this discursive move supported earlier identification and intervention for cognitive difficulties in older adults, it also worked to solidify dementia as a distinct object of investigation. Despite real differences in etiology, trajectory, and manifestation across the diseases that cause dementia, the category brought these heterogeneous experiences together within a single framework through which they could be observed, classified, measured, researched, managed, and so on. Through discursive processes of nominalization (Fairclough 2003), dementia became a coherent clinical object (a noun) which has shaped how diverse changes in cognition, behavior, and function are interpreted and acted upon in practice.

The formalization and widespread adoption of the term *Behavioral and Psychological Symptoms of Dementia* (BPSD) further extended this classificatory logic (Draper 1999). Behaviors such as agitation, wandering, calling out, resistance to care, and striking out were increasingly understood as symptoms of dementia rather than as potentially meaningful responses to unmet needs, environmental conditions, interpersonal interactions, or expressions of agency. By positioning these behaviors as manifestations of an underlying condition, BPSD discourse constituted resistance as a neuropsychiatric symptom requiring clinical management. This framing created the conditions under which BWAB could emerge as a legitimate and desirable intervention because behaviors were increasingly understood as problems residing within the person with dementia rather than as phenomena arising from complex interactions among individuals, caregivers, and care environments.

The second social structure that shaped the conditions under which BWAB was conceived and circulated was the biopsychosocial framework for understanding dementia. Building on the biomedicalization of dementia that had already taken hold, this framework introduced a more integrative view that linked biological, psychological, and environmental factors to the expression of behavior. The Unmet Needs Model (Cohen‐Mansfield and Werner 1995; Cohen‐Mansfield et al. 1989) was the key biopsychosocial framework introduced at the time that shaped the landscape for BWAB. The model proposed that many behaviors associated with dementia (e.g., agitation, aggression, and resistance to care) reflect unaddressed physical, psychological, and social needs and that environmental changes (in addition to pharmacological changes) were required in order to address behaviors. This reframing softened the biomedical narrative that positioned challenging behaviors as purely symptomatic of neuropathology; nevertheless, it still maintained a clinical logic that located interpretive authority in the hands of practitioners.

The third structure is the neoliberal reorganization of LTC that intensified throughout the 1980s and 1990s, characterized by market‐based efficiency models, managerial accountability, and the fragmentation of caregiving labor (Diamond 1992; Vladeck 1980, 2003). Scholars such as Tom Kitwood (1997) responded to these reforms by advancing the discourse of person‐centered care, which resisted the depersonalization of clients and emphasized relational and moral dimensions of care. Within this context, BWAB's distinction between “task‐centered” and “person‐centered” bathing reflected a broader critique of industrialized care practices and an effort to reclaim nursing's esthetic ways of knowing (Carper 1978).

The fourth structure is the regulatory framework built to support least restraint, which had become entrenched internationally by the 1990s through policy and accreditation frameworks. These frameworks continue to position restraint minimization as an ethical and legal imperative, defining freedom from coercion as an indicator of high‐quality, rights‐based care. Across many jurisdictions, restraint minimization is institutionalized through legislation, accreditation standards, and professional codes of conduct that codify a commitment to least restraint. In Canada, for example, the Canadian Nurses Association's (CNA) *Code of Ethics* calls on nurses to use the least restrictive measures possible. This is defined as “strategies that minimize interference with individual rights while still achieving the desired health outcomes. This approach emphasizes respecting personal freedoms and autonomy as much as possible” (Canadian Nurses Association 2025, 62). Governments and regulatory bodies across provinces and territories also echo this expectation (e.g., Fixing LTC Act 2021), and contravention of least restraint values may even involve disciplinary consequences. In Australia, for example, the Aged Care Act 2024 (Australian Government 2024) and Aged Care Rules 2025 (Australian Government 2025) stipulate that restrictive measures are only to be used as a last resort, in their least restrictive form, for the shortest possible duration. In the United Kingdom, the Care Quality Commission UK (2018) similarly stipulates that “all restrictive interventions should be for the shortest time possible and use the least restrictive means to meet the immediate need” (p. 2). These frameworks, among others, reflect a global sense that restraint minimization represents both a moral and professional standard guiding nursing practice.

At the time of BWAB's inception, growing public and professional concern also surrounded the safety of frontline healthcare workers. Reports of physical and verbal assault in LTC facilities (particularly during personal care activities such as bathing) were drawing increased attention from unions, policymakers, and professional associations (e.g., Miller et al. 1989; Namazi and Johnson 1996). Although not characterized by widespread public protest, these issues were frequently discussed in nursing and gerontology forums, professional newsletters, and occupational safety reports. The concern reflected a broader societal recognition that staff injuries and burnout were escalating and that institutional approaches to dementia care needed urgent reform (Bowie 2000; Paterson et al. 2001).

Together, these social structures—biomedicine, biopsychosocial frameworks of behavior, opposition to neoliberal reform, and the regulatory frameworks of least restraint—created the conditions under which BWAB became intelligible. Together they established the moral, professional, and practical frameworks that defined how the problem of “resistance to care” in the context of dementia could be understood and addressed. By the late 1990s, it had become both possible and expected for nurse‐researchers to describe unwanted bathing as a dual problem: a symptom of disease and a shortcoming of institutional practice that could be improved through evidence‐based, person‐centered approaches. These overlapping structures influenced not only what BWAB could claim but also the way it was received.

### 4.2 Level 2: Production and Dissemination

**Figure 1:**
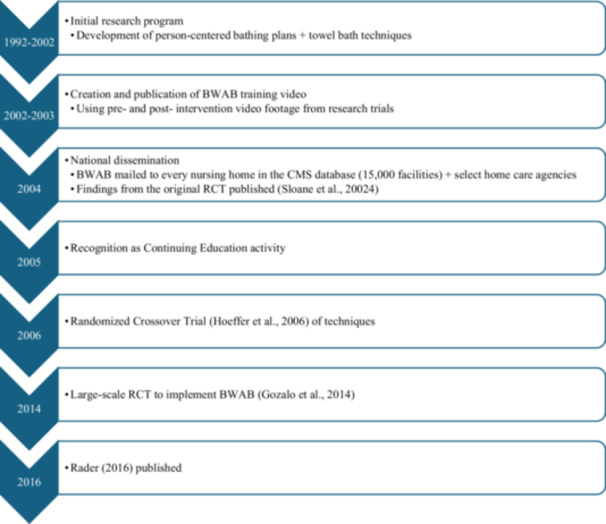
Production and dissemination timeline.

The BWAB training project is based on a series of research studies about alternative bathing techniques that occurred over 10 years (1992–2002). Two research teams identified bathing techniques to reduce agitation and aggression during bathing over the study period, which culminated in the publication of the BWAB video for widespread dissemination in 2003. The approaches trialed by the research team were called “person‐centered bathing plans” and “towel‐baths” (Sloane et al. 2004). BWAB used video footage from the original studies on these two approaches to show interactions between nursing home staff and clients before the techniques were introduced and again after. Continuing education credits were approved by various regulating bodies for frontline staff upon training completion (Calleson et al. 2006). A copy of the training video was mailed to every nursing home in the Centers for Medicare and Medicaid Services database (over 15,000 homes) at the time of publication. It was also mailed to interested home care providers, assisted living facilities, healthcare ombudsmen, and policymakers (approximately 2000 additional recipients) (Calleson et al. 2006). The findings from the original randomized control trial (RCT) on which the video was based were then published in 2004 (Sloane et al. 2004). That publication has since had a substantial influence on the field, being cited over 260 times, ranking in the 96th percentile for citations among comparable articles, and carrying a field‐weighted citation impact of 4.7 (accessed via Scopus January 21, 2026). The approaches were trialed again in 2006 (Hoeffer et al. 2006) and 2014 (Gozalo et al. 2014). Hoeffer et al. (2006) completed a randomized crossover trial comparing frontline nursing staff's approaches to both interventions and found that clients were significantly less agitated at bath time when person‐centered approaches were used. In their randomized crossover trial with 240 nursing home clients, Gozalo et al. (2014) reported widespread changes in how bathing was performed and found a statistically significant reduction in client agitation and aggression after the intervention. In 2016, in an editorial piece that proposed to take a “fresh” look at the program, one of the primary researchers on the project suggested that the program continues to be “one of the most powerful tools for promoting the need for culture change to direct care staff and administration” (Rader 2016, para. 4). Together, these studies and dissemination efforts positioned BWAB as available, practical, empirical, and morally just enough to uptake. The full production and dissemination timeline is described in detail in Figure 1.

### 4.3 Level 3: The Text

This analysis focuses on three interrelated textual dimensions: genre, discourse, and style. First, we examine BWAB's hybrid genre, which merges the authority of scientific research with the accessibility of an instructional training video, producing what Fairclough (2003) describes as a *genre chain*. A genre chain occurs when two different genres are yoked together; in this case, the genre chain links academic research with the do‐it‐yourself genre. Second, we consider the discourses that structure the video (particularly those of cleanliness and compliance) and how they define resistance as a problem requiring professional intervention. Finally, we analyze style, focusing on how nurses are inculcated into the role of the *good nurse* through the video's repeated and affectively charged emphasis on the *pleasure* of bathing.

#### 4.3.1 Genre

Individual texts belong to genres, which provide overarching structure to a text. Genres pre‐exist individual texts and organize how the texts within them are produced, interpreted, and taken up (Fairclough 2003). Genres can also play a role in regulating social relationships. For example, workplace policies do not merely convey information but authorize particular hierarchies, responsibilities, and forms of conduct. Genres may also link together to form what Fairclough (2003) terms *genre chains*, which occur when two genres are yoked together, and the governing capacity of one genre is transmitted to another (Eng and Jarvis 2020). In the case of BWAB, we see instructional texts drawing on the conventions of academic research that enhance its legitimacy and persuasive force (Eng and Jarvis 2020). As shown in Table 1, BWAB is situated at the intersection of academic research and instructional “how‐to” genres. The narrator draws on the conventions of academic research in order to bolster the credibility of the new approaches to bathing they introduce and teach to viewers. While instructional genres often democratize knowledge, BWAB reasserts professional hierarchy by positioning the nurse as the knower and the CNA as the doer.

**Table 1:** Genre conventions in BWAB.

Genre	Textual evidence	Function
Academic research	References by video narrator to “10 years of research” that backs the approaches outlined; citing “surveys” and “real life encounters” as data that informed the development of the bathing techniques the video demonstrates; use of pre/post video footage of “real care encounters” as empirical evidence to support new approaches to bathing; quotations from care aides framed as qualitative findings supporting the ease of implementing the new approaches	BWAB borrows rhetorical strategies and evidentiary conventions of scholarly writing to legitimize claims; positions the nurse‐narrator as a researcher with epistemic authority
How‐to/instructional	Step‐by‐step demonstrations (e.g., practical demonstration of equipment); contrasting “wrong” vs. “right” practice; rhetorical questions prompting reflection	Offers accessible, procedural guidance for frontline workers (who are constructed as the “doers” contrasted with the RN‐Researcher “knowers”); supports skill acquisition and behavior change
Chain	References to “surveyors issuing citations” for those who do not practice in evidence‐informed ways; statements that administrators must “give a clear message to reluctant staff to work towards change or leave”; emphasis on practicing in accordance with new knowledge	Allows the introduction of soft regulatory pressure into an ostensibly educational text; communicates policy expectations through the voice of instruction

The formation of a genre chain with academic research is important to the success of BWAB as an instructional text that enables instruction to be delivered from a position of elevated authority and facilitates what governmentality scholars describe as “governance at a distance” (Rose and Miller 1992, 173). Although BWAB does not function as formal policy, it embeds regulatory and moral expectations within an educational format. References to surveyors issuing citations and directives that administrators must require staff to “work towards change or leave” introduce compliance imperatives without explicitly invoking policy. By chaining instructional guidance with research authority, the video operates as a soft disciplinary technology: it defines acceptable care, frames resistance as a failure of evidence‐informed practice, and encourages self‐regulation among care aides while simultaneously positioning administrators as monitors of compliance.

#### 4.3.2 Discourse

From an analysis of the text's overall structure, Fairclough (2003) suggests moving into discourse analysis by examining how lexical choices, grammatical constructions, and clause ordering reflect broader worldviews. Following McCloskey's (2008) recommendation to begin discourse analysis by tracing all references to the social issue under study, we coded each mention of bathing and analyzed the modifiers and patterns surrounding it.

The video presents two types of bathing: “task‐centered bathing” and “person‐centered bathing.” Both approaches are framed as means of achieving the overarching goal of keeping people clean. Although the research team initially coded “clean” and bathing together, closer reflection revealed that the concepts were hierarchically related rather than synonymous. As outlined in Table 2, the text positioned task‐centered and person‐centered bathing as alternative approaches to the broader goal of keeping people clean. The narrator's insistence that forced bathing was necessary to “keep people clean and healthy,” followed by the claim that “there are ways to keep people clean without a battle,” reinforces the central problem as one of cleanliness rather than choice. Throughout this framing, clients' distress is treated as an avoidable consequence of poor technique rather than an expression of agency. The before‐and‐after footage strengthens this interpretation: resistance is depicted as something produced by cold water, poor setup, or caregiver error rather than as a meaningful choice.

**Table 2:** Cleanliness and biomedical discourse in BWAB.

Discourse	Examples of textual features	Framing of people living with dementia	Framing of nursing staff	Implications
Cleanliness discourse	Hyponymic pattern: Task‐centered and person‐centered bathing positioned as hyponyms of “keeping people clean” (e.g., “ways to keep people clean without a battle”; “changing the way people are kept clean”). Cleanliness is the superordinate goal; different bathing approaches/products are alternative methods to achieve an unquestioned end	Residents are implicitly positioned as indirect objects of a cleanliness project. They are the ones who must be “kept clean,” rather than agents who might legitimately decide *not* to pursue cleanliness. Little linguistic room to refuse/reject cleanliness as autonomous people	Nursing staff are framed as responsible for choosing the best method of achieving a fixed goal (cleanliness)	The central question becomes *how* to bathe someone who refuses, not whether they should be bathed at all; unwanted care is normalized as inevitable or at least technique‐dependent. Resistance is handled by shifting the means, not reconsidering the end
	Transitivity pattern: Cleanliness serves as the direct object: “to keep/to get people clean” (e.g., “ways to keep people clean,” “no‐rinse soap gets people just as clean”)			
	Reported genericized speech (“people often ask ‘does no‐rinse soap really get people clean?'”)			
Biomedical discourse (dementia‐as‐pathology)	Direct testimonial from a Registered Nurse: “All of the residents on our units have dementia, we believe that they still have the right to make choices and say no, if they don't want a bath. However, the CNAs are very good at coming up with ways to keep them clean, and to wash them up, that they can tolerate.”	Residents are acknowledged as having a right to say no to a bath but not to refuse cleanliness itself, creating a constrained, conditional autonomy	Nursing staff must decode and reframe refusals	Refusal is procedurally recognized but substantively overridden; the work of staff is to convert “no” to a form of cooperation that satisfies cleanliness goals
	Narrator's commentary on before and after videos of baths: “How staff communicate with residents before and during the bathing process is really important. You saw in an earlier video clip that when the aide used the word bath, the resident said she didn't want a bath. And then the CNA skillfully said, ‘Well, we won't give you a bath we'll give you a wash up’ and then that was fine with the resident.”			

The foreclosure of clients' agency also stands out in the transitivity patterns in the text. Throughout the video, the verbs *to keep* and *to get* take cleanliness as their direct object and clients as indirect objects (“ways to keep people clean,” “changing the way people are kept clean,” and “does no‐rinse soap really get people clean?”). Staff become the grammatical agents responsible for “keeping” and “getting” of cleanliness, while clients become the bodies to whom cleanliness is delivered. Grammatical constructions that would position clients as agents (e.g., to refuse/avoid/reject cleanliness)—while linguistically possible—are absent from the video and highlight the deferential role of people living with dementia.

A suppression of autonomy is reinforced by the social structure of biomedicine and the related discourse of dementia‐as‐pathology. This discourse construes people living with dementia as having lost the capacity for self‐knowledge, coherent desire, or authentic self‐expression (Morris et al. 2024). The notion that dementia erodes the true self quietly underpins the video's approach insofar as refusals are never treated as genuine expressions of will. Instead, they interpretable as misunderstandings to be corrected, sensory discomforts to be minimized, or behavioral disturbances to be managed and worked around. Cleanliness discourse, in this sense, aligns seamlessly with pathologizing discourses that undermine the credibility of clients' emotional and embodied communication and cast people living with dementia as unreliable narrators of their own desires. If a client's *no* is presumed to reflect cognitive impairment rather than authentic refusal, then the “skillful” redirection (Barrick et al. 2003), reframing, and “creative” (Barrick et al. 2003) workarounds outlined in Table 2 become intelligible as care rather than coercion. This discursive framing also prepares the ground for a different layer of analysis: the stylistic resources through which nurses' identities are shaped and their actions morally justified in the text. This language makes visible how apparently neutral linguistic choices organize bathing around an unquestioned cleanliness imperative, systematically positioning clients as objects of care that is required because of clients' underlying pathology and staff as responsible agents who treat that pathology.

#### 4.3.3 Style

Having established how BWAB organizes bathing through cleanliness and biomedical discourses, this section turns to style as the final step in the textual analysis. According to Fairclough (2003), style is the discursive means through which texts do their ideological work. They constitute social identities and establish the truths, values, and moral commitments associated with those identities. In this sense, texts interpellate or “hail” people as particular kinds of subjects who are expected to act, feel, and believe in particular ways (Althusser 1971). Highly stylized texts make these expectations apparent by signaling the consequences of failing to recognize or inhabit the subject positions they offer.

In BWAB, the language of pleasure operates as a key stylistic resource through which the figure of a good and morally competent nurse is produced and normalized and the figure of the happy and compliant LTC client is produced. A client's pleasure is treated as a moral benchmark against which care practices are evaluated and against which nurses are interpellated as morally just professionals. Through repeated injunctions to make bathing “pleasurable,” the text evaluates person‐centered bathing (and the nurses who practice it) as morally superior and implicitly casts task‐centered bathing (and the nurses who practice it) as morally lacking. The insistence that “bathing should be pleasurable for people in care settings, just as it is for us” (Barrick et al. 2003) is repeated throughout the text and culminates in the directive that if bathing is not pleasurable “or at least tolerable” (Rader 2003) then “staff need to adapt” (Rader 2003).

There is, however, a clear limit to the amount and kind of pleasure that assisted bathing may legitimately produce. In BWAB, the morally good nurse is not simply one who prioritizes pleasure, but one who ensures that bathing is pleasurable without becoming excessive, lingering, or transgressive. This limit is signaled linguistically through modality. The narrator's repeated softening of pleasure into what is “pleasant” or “or at least tolerable” provides upper boundaries to the pleasure clients can experience. It is a modal shift that lowers the intensity and moral demand to ensure clients' pleasure and works to reframe pleasure from an embodied and potentially transgressive experience into one of mild comfort and acceptability. The upper limits of pleasure are also conveyed visually and interactionally throughout the video footage accompanying the narration. While clients are shown smiling, sighing, or verbally expressing comfort, staff remain quiet, efficient, and emotionally restrained. They rarely linger or reciprocate in ways that might suggest shared enjoyment. Attenuated and acceptable pleasure is thus carefully located in the client's body, rather than in relational intimacy or mutual sensation. This stylistic containment of pleasure reflects historical traditions in which bathing has been tied to ideals of cleanliness, morality, and whiteness (Carroll 2023; Ore 2021; Sandlin and Maudlin 2015). More overt or modally strong forms of bodily pleasure have historically been displaced onto spaces and subjects marked as deviant or taboo (Ferris 2021; Morgan 2015; Okello 2025). Against this backdrop, BWAB can be read as carefully advocating for clients' pleasure in bathing while simultaneously disciplining it. Pleasure, in this formulation, must be felt in moderation, managed by the nurse, and stripped of any meanings that might threaten institutional norms or the moral identity of the caregiver. Pleasure operates as a sort of moral alibi that legitimizes certain types of nursing care and cultivates a professional subject who provides but also constrains pleasure as a moral obligation.

## 5 Discussion

This analysis raises broader questions about how autonomy is understood and managed in residential dementia care. Within the literature on nursing ethics, autonomy is increasingly conceptualized as relational, fluctuating, and context‐dependent rather than as a fixed cognitive capacity (Bartlett 2010; Beattie 2009; Darby and Dickerson 2017; Dewing 2008; Dworkin 1986; Gasparini et al. 2021; Kitwood 1997; Serbser‐Koal et al. 2024; Svendsen et al. 2017). Despite this shift, everyday care practices across the continuum of nursing care remain governed by biomedical preoccupations with impairments and professional prioritization of risk management (Todd et al. 2021). BWAB reflects this tension clearly. While the text acknowledges clients' distress and resistance during bathing, it ultimately treats refusals as a problem to be resolved rather than a moment that might warrant rethinking whether care ought to proceed at all. In doing so, it aligns with a broader tendency in dementia care to recognize autonomy rhetorically while constraining it procedurally.

BWAB places significant pressure on nurses to find “workarounds” to clients' resistance (Rader 2003). Because the endpoint (successful bathing) is predetermined, a nurse's use of softly coercive approaches is reframed within the text as “skillful” or “creative.” In BWAB, the moral weight placed on achieving cleanliness encourages nurses to reinterpret resistance as misunderstanding, discomfort, or behavioral disturbance to be managed. This framing risks normalizing coercion as care. The incorporation of pleasure into this framework does not resolve these tensions; it reshapes them. When refusal cannot function as a reason to stop, the measure of care shifts to whether clients experience coercion as sufficiently comfortable. Pleasure becomes a metric of morally good nursing even when refusals persist. Feminist scholars have cautioned that the presence of pleasure does not negate coercion when refusal is structurally foreclosed (Ahmed 2010; Cahill 2014; MacKinnon 1989). The requirement that people living with dementia experience or display pleasure during bathing risks providing nursing staff with a moral alibi that obscures the ongoing erosion of clients' autonomy under conditions of unequal power.

These findings underscore the need for greater ethical scrutiny of how autonomy is operationalized in intimate bodily care. Relational models of autonomy emphasize negotiation, responsiveness, and creativity, but they also require attentiveness to power and the possibility that continued insistence on care may constitute harm. The expectation that nurses must always adapt until care is accepted leaves little room for recognizing persistent refusal as meaningful, or for legitimizing non‐intervention as an ethical choice. By foregrounding the moral pressures placed on nurses to resolve resistance through cleanliness, pleasure, or both, this analysis highlights the ethical costs of care models that prioritize outcomes over agency.

Within the prevailing risk‐averse healthcare system in North America, hygiene (or lack thereof) is consistently presented as a paramount danger to be managed (Brown et al. 2008). Dirt, and particularly stool that is not washed from the skin, is positioned as a threat to skin integrity and infection control. The failure of care providers to remove it is widely considered a moral failing of staff. This framing of dirt‐as‐danger is not new (see Douglas 2002), and it continues to exert a significant influence on care practices (Morris et al. 2025). It is, however, a troubling calculus that positions skin integrity as self‐evidently valuable while bodily integrity becomes negotiable. Seemingly mundane nursing care (e.g., hair washing, toenail clipping, and denture care) regularly proceeds with people living with dementia (Hamers et al. 2016) in ways that are anything but mundane. On a structural level, these violations reveal a healthcare system willing to dismiss bodily integrity in pursuit of bodily maintenance. On a human level, it reveals just how easily the subjectivity and autonomy of people living with dementia can be subordinated to institutional imperatives of cleanliness, safety, and risk reduction.

BWAB remains a seminal text in residential dementia care, shaping how bathing is taught, understood, and practiced well beyond the context in which it was originally produced. Precisely because of its enduring influence, BWAB warrants careful critical attention. More broadly, this analysis suggests that other seemingly mundane nursing interventions warrant the same. Bathing, feeding, repositioning, oral hygiene, continence care, medication administration, and other ADLs are routinely framed as straightforward acts of care. That framing has consequences. When these activities are understood as low‐skill tasks, their delegation to unregulated care providers becomes justifiable. The ethical complexity and clinical judgment embedded within them are also delegated to unregulated care providers who do not always practice within nursing codes of ethics and do not hold the same level of professional accountability. If bathing can justify coercion, restraint, sedation, and the overriding of refusal, it can no longer be understood as a simple task, and it can no longer be delegated with the same casualness. Critical examination of these activities invites not only renewed scrutiny of the interventions themselves, but of the workforce models built on the assumption that they require no scrutiny at all.

To conclude, we must note that this analysis should not be interpreted as an argument against all rebuffed hygiene care or against best‐interests‐focused decision‐making in advanced dementia. Nurses will continue to encounter situations in which concerns regarding skin integrity, infection, and safety must be carefully balanced against respect for autonomy. This analysis also does not provide a framework for determining the authenticity, meaning, or legitimacy of refusal. Rather, it examines the discursive conditions that shape how refusals come to be understood, justified, and acted upon in dementia care and invites critical reflection on the ethical consequences of treating bodily autonomy as continually negotiable and resistance as something to be managed rather than engaged.

## Ethics Statement

The authors have nothing to report.

## Conflicts of Interest

The authors declare no conflicts of interest.

## Permission to Reproduce Material From Other Sources

Permission to reproduce material from other sources was not required, as no copyrighted images or figures were reproduced in this manuscript.

## Use of AI

Generative AI tools were used to support brainstorming, editing, and refinement of manuscript text. The authors retained full responsibility for all conceptual development, analysis, interpretation, and final content decisions. All AI‐generated suggestions were critically reviewed, revised, and verified by the authors prior to inclusion.

## Data Availability

The data that support the findings of this study are available from *Bathing Without a Battle*. Restrictions apply to the availability of these data, which were used under license for this study. Data are available from https://bathingwithoutabattle.unc.edu/ with the permission of *Bathing Without a Battle*.
